# fMRI evidence that hyper-caricatured faces activate object-selective cortex

**DOI:** 10.3389/fpsyg.2022.1035524

**Published:** 2023-01-12

**Authors:** Ryan Elson, Denis Schluppeck, Alan Johnston

**Affiliations:** School of Psychology, University of Nottingham, Nottingham, United Kingdom

**Keywords:** faces, object-selective cortex, face space, fMRI, caricatured faces, PCA

## Abstract

Many brain imaging studies have looked at the cortical responses to object categories and faces. A popular way to manipulate face stimuli is by using a “face space,” a high dimensional representation of individual face images, with the average face located at the origin. However, how the brain responds to faces that deviate substantially from average has not been much explored. Increasing the distance from the average (leading to increased caricaturing) could increase neural responses in face-selective regions, an idea supported by results from non-human primates. Here, we used a face space based on principal component analysis (PCA) to generate faces ranging from average to heavily caricatured. Using functional magnetic resonance imaging (fMRI), we first independently defined face-, object- and scene-selective areas with a localiser scan and then measured responses to parametrically caricatured faces. We also included conditions in which the images of faces were inverted. Interestingly in the right fusiform face area (FFA), we found that the patterns of fMRI response were more consistent as caricaturing increased. However, we found no consistent effect of either caricature level or facial inversion on the average fMRI response in the FFA or face-selective regions more broadly. In contrast, object-selective regions showed an increase in both the consistency of response pattern and the average fMRI response with increasing caricature level. This shows that caricatured faces recruit processing from regions typically defined as object-selective, possibly through enhancing low-level properties that are characteristic of objects.

## 1. Introduction

In regular social interactions, we may encounter hundreds of faces every day. Most human observers can rapidly recognise the identity (Ramon et al., 2011), process the emotion (Leppänen and Hietanen, 2004), or form an impression of a person or their intentions (Bar et al., 2006; Willis and Todorov, 2006; Sutherland et al., 2013) from visual information alone.

It is estimated that humans know on average around 5,000 faces (Jenkins et al., 2018) but despite much research, it is largely unknown how we encode all those familiar faces, in addition to all unfamiliar ones. Face space (Valentine, 1991; Valentine et al., 2016), an influential account of face representation, has been widely used to study the neural representation of faces in humans (Loffler et al., 2005; Carlin and Kriegeskorte, 2017) and non-human primates (Leopold et al., 2006; Chang and Tsao, 2017). The idea has also found application in automatic face recognition systems (Sirovich and Kirby, 1987; Turk and Pentland, 1991; Zhu et al., 2013; Deng et al., 2014). Caricatured face images that deviate substantially from average, including artistic caricatures, evidently amplify characteristic features of faces. But it is unclear if and how face space is represented in the brain and what the exact neural representation of faces distant from the average face might be.

In *face space*, a multidimensional space with a representation of an average face at the origin, individual face *exemplars* are thought of as points (at a certain distance and direction with respect to the origin). The dimensions of this space could be derived from discrete, descriptive changes in the shape or position of features (e.g., the distance between the eyes or the width of the mouth). Alternatively, the dimensions may reflect more abstract and global descriptors of shape and texture.

In a face space representation, individual *identities* correspond to a given direction relative to the origin. The distance from the origin indicates how different a particular face is from the average. Faces whose representation is located a greater distance from the average are expected to generate stronger responses from the population of neurons sensitive to the given identity’s facial properties. This idea of “norm-based” coding, coding relative to the average or norm, has received strong supporting evidence (e.g., see Leopold et al., 2001; Anderson and Wilson, 2005; Jiang et al., 2006, 2007; Rhodes and Jeffery, 2006; Webster and MacLeod, 2011; Little, 2012; Chang and Tsao, 2017). Neurons representing facial information could form a basis to span this space, rather than being tuned to a particular identity. The projections of a face onto a set of basis neurons may code the different identities (Chang and Tsao, 2017) in terms of the relative firing rates of this population of neurons.

The neural basis of norm-based coding has recently been clarified by new research in macaques (Koyano et al., 2021). Rhodes and Jeffery (2006) proposed that norm-based coding was based on two opponent channels with the average face activating each equally. The opponent channels can be associated with ‘axis coding’ that shows monotonic ramp-tuning through the norm in single-cell recordings (Chang and Tsao, 2017). Norm-based coding also gives rise to V-shaped coding (e.g., Freiwald and Hosoya, 2021) whereby there is a minimum response to the norm relative to more peripheral faces, regardless of direction. V-shaped coding was first demonstrated at a single cell level and at a population level by Leopold et al. (2006). Recently, evidence has shown that both mechanisms are present in the same set of individual neurons, with axis coding occurring approximately 100 ms before V-shaped coding (Koyano et al., 2021). However, the V-shape was driven by a decrease in the firing rate to average faces, likely from lateral inhibition resulting from synchronous firing across the population to the average face (Koyano et al., 2021). Whilst axis coding supports the initial coding of the neuron, V-shaped responses reflect a consequence of many neurons firing to the average face in synchrony.

Chang and Tsao (2017) show that rather than responding to specific identities, neurons in areas ML/MF of the macaque temporal lobe (middle lateral/middle fundus) and the more anterior AM (anterior medial) responded to combinations of shape and texture information. Firing rates increased linearly with the magnitude of a face’s projection onto the neuron’s preferred dimension or ‘axis’ of change, but only in the preferred direction of change; face stimuli along the same axis but on the opposite side of the mean decreased the neuron’s firing rate. Variations in facial appearance orthogonal to a neuron’s preferred dimension, however, did not change its firing rates. This invariance to changes along orthogonal axes may explain the lack of an aftereffect to faces that lie on a different trajectory from the adapting stimulus (Leopold et al., 2001; Anderson and Wilson, 2005; Rhodes and Jeffery, 2006). From a theoretical standpoint, it allows face processing to be based on a highly efficient calculation (linear projection), requiring relatively few neurons to encode a very high-dimensional face space. Interestingly, it has also been found that faces activate a more broadly-based representation within an object space. Recent work has shown that faces may be situated in the animate, ‘stubby’ quadrant of the identified 2D (animate/inanimate and stubby/spiky) space, although many aspects of this representation remain unknown (Bao et al., 2020).

Because faces are more densely clustered around the mean, those further from average should appear more distinctive (Valentine et al., 2016). This idea is supported by evidence showing that caricatures are rated as more distinctive than their veridical face or anti-caricature (Lee et al., 2000). If the dimensions are ordered in terms of the amount of facial variance they encode, then more distinctive faces may also load more onto less prevalent dimensions of variation, in which case direction in the space may also reflect distinctiveness (Hancock et al., 1996). The direction and distinctiveness in face space not only impacts recognition, but also the first impression that is attributed to that face (Olivola et al., 2014; Over and Cook, 2018), and can indicate poor childhood health or genetic disorders (Rhodes et al., 2001; Gad et al., 2008; Babovic-Vuksanovic et al., 2012; Dolci et al., 2021).

Faces can also be made artificially more distinctive through caricaturing. Caricatures, versions of ‘veridical’ face images that can be derived from extrapolations in face space, enhance behavioural performance over veridical faces, suggesting they may elicit stronger responses in the brain. Caricaturing line drawings and photographs enhances recognition (Rhodes et al., 1987; Mauro and Kubovy, 1992; Lee et al., 2000;Kaufmann and Schweinberger, 2012; Schulz et al., 2012), whilst anti-caricaturing (making the stimuli more average) leads to longer reaction times (Rhodes et al., 1987; Schulz et al., 2012) and reduced identification accuracy (Lee et al., 2000). Interestingly, caricaturing even improves recognition accuracy in deep convolutional neural networks (Hill et al., 2019). Subsequent recognition of veridical faces is enhanced by caricaturing during encoding (Rodríguez et al., 2009), suggesting that exaggerating the features or configuration can help create representations for new faces. Furthermore, adapting to caricatures makes veridical images appear more average (Carbon and Leder, 2005), consistent with the idea that the subset of neurons processing caricatured faces are the same as for their veridical versions. Caricaturing exemplars from the norm also increases the EEG amplitude of the face-selective N170 and N250 ERP responses (Kaufmann and Schweinberger, 2012; Schulz et al., 2012), although other neural responses such as the P200, decreased with distance from average (Schulz et al., 2012), suggesting that some neural processes may encode averageness and typicality.

Studies investigating distance from average on the neural response have adopted a variety of methods making direct comparison difficult (Loffler et al., 2005; Leopold et al., 2006; Susilo et al., 2010; Davidenko et al., 2012; McKone et al., 2014; Carlin and Kriegeskorte, 2017; Chang and Tsao, 2017). Chang and Tsao (2017) found near-linear increases with increasing distance through the average in macaques using single unit recordings, as has prior research (Leopold et al., 2006; which included moderately caricatured faces). Likewise, some behavioural work in humans using adaptation has found that the strength of the aftereffect caused by adapting to faces with varying eye and mouth height increased linearly, even outside the range of natural variability (Susilo et al., 2010). Other research suggests that the strength of identity aftereffects following adaptation increases linearly, but then is slightly reduced but constant past the ‘naturalness boundary’ (McKone et al., 2014). Results from functional magnetic resonance imaging (fMRI) studies have found saturating responses to stimuli at a certain distance from average (Loffler et al., 2005; Carlin and Kriegeskorte, 2017; see pg. 1,387). The faces in these studies did not extend far past the range of natural plausibility.

Electrical brain stimulation of the fusiform face area (FFA; Kanwisher et al., 1997) produces metamorphosis of viewed faces (Parvizi et al., 2012), suggesting that hyperactivity in the FFA delivers the perception of a caricatured face and thus may represent distance in face space. The perceived change in shape is consistent with suggestions that the FFA is homologous to the area ML in macaques (Tsao et al., 2003, 2008; note the 2003 paper refers to area ML as macaque area pSTS) given that this region shows greater sensitivity to shape over texture (Chang and Tsao, 2017). There is debate, however, over exact homology between human and macaque face processing systems (Yovel and Freiwald, 2013; Rossion and Taubert, 2019).

**Figure 1:**
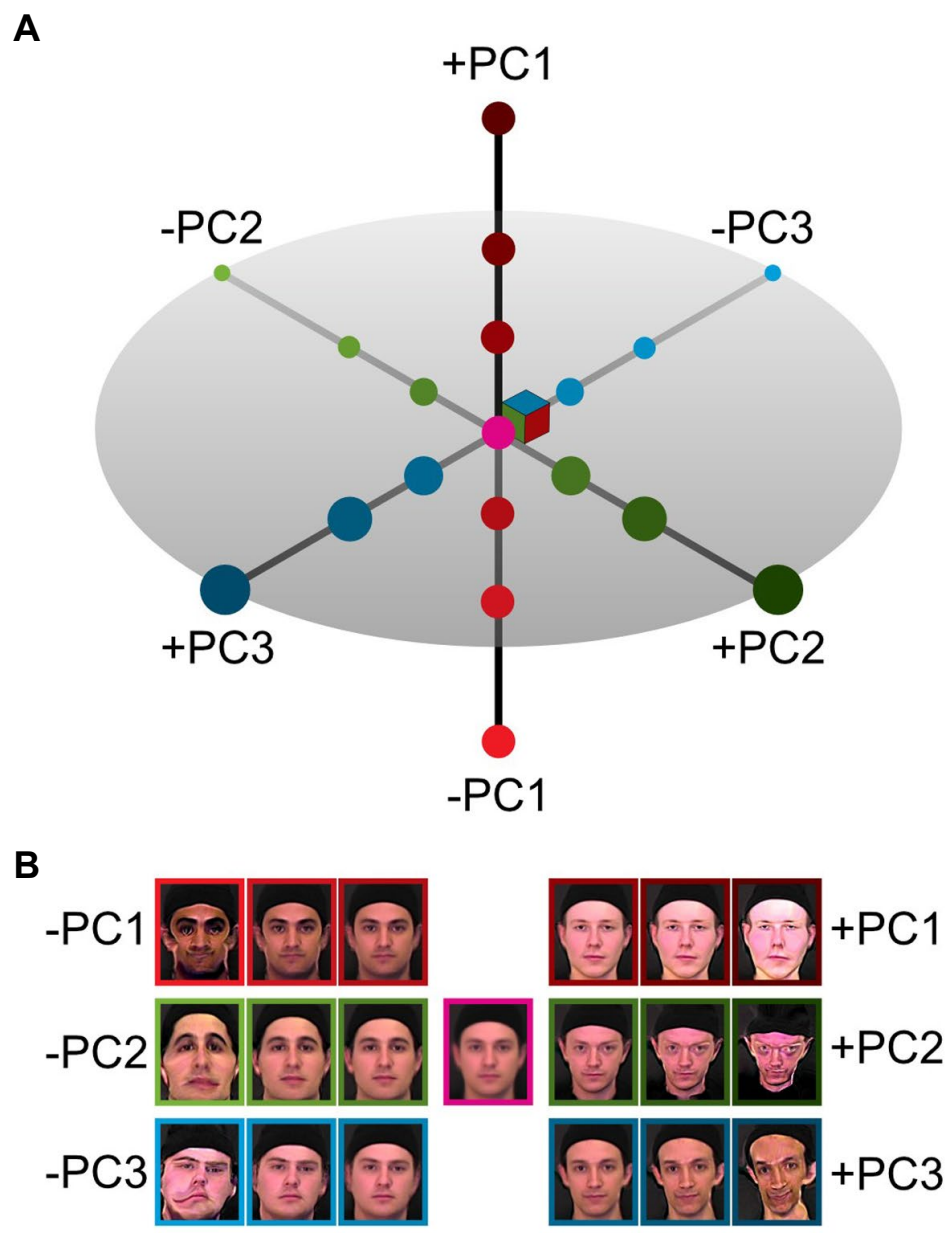
Caricaturing in face space. **(A)** An illustration of the three major axes of a principal component face space constructed from images of male faces. The origin in this space corresponds to the average face. Principal components (PC1 red, PC2 green, PC3 blue) are ordered by variance explained in the underlying data. **(B)** Example images created by modulating each of the principal components independently. Positive and negative deviations from the origin result in opposing changes in reconstructed images, increasingly caricatured with larger distances from the origin (average face, centre).

Hyper-caricatures, images that appear distorted beyond the range of natural appearance, can be generated by extrapolating in face space. In a face space constructed by principal component analysis (PCA), using weights much larger than those corresponding to typical faces shifts the representation further from the mean (see Figure 1). This allows the generation of a parametrically controlled set of realistic and hyper-caricatured faces that can be used as stimuli for brain imaging. Specifically, we wanted to explore how the blood-oxygen dependent (BOLD) fMRI signal changes in face-selective cortex, including the FFA, with stimuli at various distances from average in face space and with concomitant changes in perceived naturalness.

We hypothesised that there would be an increase in the BOLD response amplitude in the FFA and other face-selective areas with increases in caricature level. To summarise the experimental design, participants first undertook a behavioural session in which they identified the point along different directions in the PCA space where the face stimuli switch from appearing natural to caricatured. The caricature level of stimuli for the fMRI session were then chosen to straddle those perceptual boundaries: some stimuli appeared closer to average and natural, whilst others appeared hyper-caricatured. Stimuli were presented in an event-related design to avoid adaptation to a specific axis (Loffler et al., 2005; Davidenko et al., 2012). Inverted (upside down) stimuli were also presented to identify low-level effects of increased caricaturing (Davidenko et al., 2012). Inverted faces contain the same low-level properties as their upright counterparts, but have been shown to decrease the fMRI response in face-selective areas (Yovel and Kanwisher, 2004, 2005; Nasr and Tootell, 2012; James et al., 2013). We therefore considered that the effect of caricature level might be greater for upright faces than inverted faces.

Our results show that in the right fusiform face area (FFA), *the patterns of fMRI response* were more consistent as caricaturing increased. However, we found no consistent effect of either caricature level or facial inversion on the average fMRI response in the FFA or face-selective regions more widely. Therefore, we also explored the response in object and scene-selective areas. In contrast to face-selective regions, object-selective regions showed an increase in both the consistency of response pattern as well as average fMRI response with increasing caricature level.

## 2. Materials and methods

### 2.1. Participants

Nine healthy, neurologically intact volunteers with normal or corrected-to-normal vision were recruited for this study. Participants were aged between 22 and 36 years old (mean = 27 years, 6 months, SD = 4 years, 1 month). Three were female, six were male. No other demographic details were collected. The sample was a mix of postgraduate research students and staff from the School of Psychology at the University of Nottingham, recruited through a mix of convenience and snowball sampling. All participants gave fully informed consent and were screened for any MRI contraindicators before taking part in the experiment. The study was approved by the School’s ethics committee.

### 2.2. Apparatus

The experiment was built in MATLAB version 9.5 (R2018b) using the Psychophysics Toolbox extensions (Psychtoolbox-3 version 3.0.17; Brainard, 1997; Pelli, 1997; Kleiner, 2007). The behavioural experiment was run on a 13” MacBook Pro (1,280 × 800 pixels). Participants responded solely through moving and clicking the mouse. Viewing distance was approximately 60 cm. For the MRI experiment, stimuli were presented on a 32″, 1,920 × 1,080 pixels BOLDscreen32 (CRS Ltd., Rochester, Kent) with a refresh rate of 120 Hz at the back of the bore through a mirror mounted on the head coil. Viewing distance was approximately 120 cm.

### 2.3. Stimuli

Stimuli were made using two separate PCA spaces, one derived from 50 images of female faces and another from 50 male faces. The input images were all aligned using the positions of the eyes and then warped to the average of the faces using the Multi-channel Gradient Model (Johnston et al., 1992, 1999), providing shape-free textures as well as the x and y warp information to convert the texture of the face back to the individual’s facial shape. The x-y warp fields were appended to the shape-free textures and PCA was performed on these full warp-texture vectors using a procedure described by Nagle et al. (2013). The PCA extracts texture and shape covariations and maps these commonalities into an orthogonal space. Face images can be reconstructed by taking the texture for a given position of the PCA space, and spatially displacing the pixels by the distances contained in the corresponding x-y warp fields (see Supplementary Figure S1). Reconstructed stimuli were 100 pixels wide by 120 pixels high. In the MRI experiment, the stimuli were feathered into the RGB background around the edges.

To create the stimulus set for the experiment, the first 5 components in each of the PCA spaces were manipulated. The PCA returns eigenvectors of unit length. It also returns values of how the input images load onto each of the components. The components in our space were scaled by 1 standard deviation (SD) of the loadings, such that moving 1 ‘unit’ along a given component reflected a change of 1 standard deviation of the loadings of the input set on that component.

### 2.4. Behavioural task

To establish the caricature levels at which faces turned from natural (physically plausible) to unnatural (physically implausible), we performed a behavioural experiment outside the scanner. This also helped to familiarise participants with the stimuli.

Stimuli scaled the first five components of each gender’s PCA space in both the positive and negative directions (20 possible stimulus directions: 2 gender *5 PCs *2 directions), with each unique trial type presented 6 times in a random order – 120 trials in total. Stimuli were presented centrally on a grey background at half the screen height (approximately 11.2^o^ of visual angle).

Using a method of adjustment, participants identified the transition points to unnatural stimuli by moving a mouse. Stimuli were dynamically updated at a caricature level controlled by the horizontal position of the mouse. A red dot on a scale bar served as a visual cue. Before each trial, an animation showed the full range of possible caricaturing for that trial (see Supplementary Figure S2A, for demonstration videos see Supplementary materials). Participants confirmed their choice with a mouse click and the next trials started after a 1,000 ms inter-stimulus interval. Because some components lead to distortions faster than others, the caricaturing applied to the stimuli was based on some pilot results from 5 independent participants (see Supplementary Table S1). Randomly varying the maximal amount of caricaturing on each trial prevented the slider’s position being used to indicate the boundary for the given component.

No fixation cross was presented so participants could freely explore the faces, and there was no time limit. Breaks were provided every 40 trials. On average participants took approximately 30 minutes to complete the experiment.

For each participant, the average naturalness boundary for each component was calculated by taking the mean transition point across the 6 repetitions. The value of this position on the scale translated to the number of standard deviations (in terms of the loadings of the input set onto the PCA space) from the origin of the space. The results of the first 7 participants were used to scale the stimuli for the MRI experiment (see Supplementary Figure S3; Supplementary Table S2). Results of all participants can be seen in Supplementary Table S3.

### 2.5. MRI study

#### 2.5.1. Localiser and caricature scans

The fMRI study consisted of two sets of scans. To find cortical regions responding to various categories of stimuli, we ran a standard functional localiser experiment using a randomised block design. We also ran a set of event-related scans in which individual images of test stimuli were presented (“caricature scans”).

In the functional localiser, images of faces, scenes and objects were presented in a block design. Each block consisted of 8 images from one category. Face stimuli included photographs of 24 different identities (12 male, 12 female) taken at frontal pose, and 45^o^ rotated in yaw in either direction. Not all views of each identity were presented. Images of scenes included both natural and manmade scenes, including pictures of buildings, both from the inside and outside. Objects included both manmade and natural objects. Faces and objects were presented on greyscale masks to occupy the same space as the scene stimuli (see Supplementary Figure S4). All stimuli were presented centrally and extended to approximately ± 8° of visual angle. Each stimulus was presented for 1 s with no ISI, with 8 s between blocks giving an 8 s ON, 8 s OFF sequence. The experiment began and ended with 8 s OFF. During the localiser a simple attention task was used: a black fixation cross was presented centrally throughout which 130 times within a scan turned red for 50 ms and participants had to respond by pressing any button on the button box. Any response within 1.5 s was classed as a hit. Each run of the functional localiser took 6 min and 32 s.

**Figure 2:**
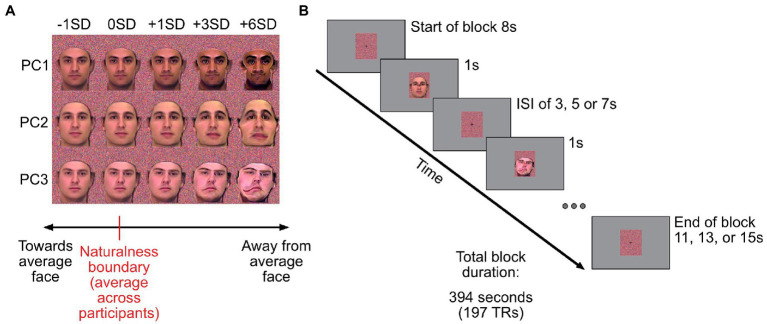
Caricatured stimuli used and outline of the fMRI experiment. **(A)** Example images corresponding to the five scaling levels used in the MRI experiment. First column of images: stimuli that are 1 standard deviation (SD) closer to the average face. Second column: group-averaged naturalness boundary for a given component (0SD). Other columns: images corresponding to +1, + 3 and + 6SD away from average. Stimuli were presented on colour masks (Gaussian noise on each R, G, B channel, with mean and standard deviation derived from the face stimuli). **(B)** Timings for trials in the event-related caricature scan. The experiment started and ended with 8 s of fixation (dynamically changing coloured masks only). Stimuli were presented for 1 s, followed by a variable inter-stimulus interval of 3, 5 or 7 s. To control for attention, participants had to report colour changes of the fixation cross (black to red), which occurred randomly 42 times within each run. The background colour mask changed dynamically every second.

During the caricature scan participants were presented with stimuli created by modulating the first three components of the male PCA space from the behavioural study. Using the averaged naturalness boundaries from the behavioural experiment, participants were shown faces that corresponded to the mean (across participants) naturalness boundary (0SDs), one SD (across participant responses) closer to the average face (−1SD), or one, three or six SDs further away from the average (see Figure 2A).

In each run, participants were presented with 5 caricature levels for each component (the average naturalness boundary plus −1, 0, +1, +3, and + 6SDs). Picture plane inverted images of the most (+6SDs) and least caricatured (−1SD) face stimuli were also presented. This provided 21 unique stimuli per run (15 upright and 6 inverted) which were repeated 3 times each in a run. The experiment started with 8 s of rest. Subsequently stimuli were presented for 1 s with a variable ISI of either 3, 5, or 7 s, with equal numbers of each ISI duration across each run. Trial timings can be seen in Figure 2B. The order of stimuli and ISI durations was pseudorandomised across runs. To ensure all runs were the same duration, the final stimulus of each run was always followed by the remaining ISI and a further 8 s of rest (minimum of 11 s in total) to allow for the lag in the haemodynamic response. Each run lasted 6 min and 34 s. As in the localiser scan, participants responded when the centrally presented fixation cross turned red. This occurred 42 times during the run.

#### 2.5.2. Data acquisition

For the localiser, functional data were acquired across 2 block-design runs, each lasting 392 s (196 volumes), one at the start of the scanning session and one at the end. Caricature scans were acquired across 3 event-related runs (4 for one participant), each lasting 394 s (197 volumes).

Data were acquired on a 3 T MRI scanner (Phillips Achieva) at the Sir Peter Mansfield Imaging Centre at the University of Nottingham using a standard 32 channel head coil. Functional (BOLD) images were acquired with 2D gradient echo EPI sequence (multiband 2, SENSE *r* = 1). Parameters were TR/TE 2000 ms/32 ms, FA 77°. There were 34 axial slices; voxel size was 2.4 × 2.4 × 3 mm, 80 × 80 voxels per slice. High-resolution T1 MPRAGE structural images were obtained with the following parameters: TR/TE 8.1 ms/3.7 ms, 1 mm isotropic voxels, 256 × 256 voxels, FOV = 256 × 256 mm, 160 sagittal slices.

#### 2.5.3. Data analysis

We used a combination of tools to analyse fMRI data: mrTools (Gardner et al., 2018) and custom MATLAB code, as well as FreeSurfer (Fischl, 2012) for cortical segmentation and anatomically defined regions of interest and FSL (Jenkinson et al., 2012) for spatial smoothing and mask dilation. Analyses were performed in individual participant space.

#### 2.5.4. Anatomically restricting the analyses

We focused our analysis on the occipito-temporal cortex, bilaterally, including the FFA, the OFA (occipital face area; Puce et al., 1996; Halgren et al., 1999) and pSTS (posterior superior temporal sulcus; Morris et al., 1996, 1998). We defined larger anatomical ROIs from FreeSurfer parcellations to span the majority of the occipito-temporal cortex, spanning both hemispheres (combining ‘lateraloccipital’, ‘fusiform’, ‘inferiortemporal’, ‘middletemporal’, ‘superiortemporal’, ‘bankssts’, ‘supramarginal’ and ‘inferiorparietal’ ROIs from the Deskian/Killiany atlas). ROIs were created by converting the parcellation labels into volumetric masks (FreeSurfer: *mri_annotaion2label* and *mri_label2vol*) and dilated using a single pass of a 3-voxel box kernel to fill any holes (*fslmaths*).

#### 2.5.5. Pre-processing

The caricature and localiser scans were first motion corrected within and between scans in mrTools (Gardner et al., 2018) using the mean volume of the second caricature scan (mid-point of the scanning session) as a reference frame. Motion correction used linear interpolation and drift correction was applied. The motion corrected functional runs were then spatially aligned to the participants’ anatomical scans. The localiser data was spatially smoothed (3D Gaussian, FWHM 5 mm). For the caricature data, voxelwise data was extracted from the face, object, and scene-selective ROIs. For the univariate analysis and multivariate pattern analysis on the data no spatial smoothing was applied.

For both the localiser and caricature scans, data were converted to percentage signal change by subtracting the mean intensity for each voxel across the scan, and dividing by the mean [(*x*-mean)/mean], temporally high-pass filtered (cut-off 0.01 Hz) and, for the univariate analysis, concatenated over scans, taking care to keep track to the transition points between scans. This allowed for the GLM analysis to be reframed in block matrices, requiring only one GLM per set of localiser and caricature scans.

#### 2.5.6. Defining the FFA and face-selective, object-selective, and scene-selective voxels

To define participant-specific functional ROIs, we used a GLM approach and restricted the analysis to the anatomical ROI described above. Analyses were performed in individual scan space. The 3 explanatory variables (EVs) were faces, objects and scenes, specified by 8 s ON boxcar regressors convolved with a double gamma haemodynamic response function (HRF). To define face-selective areas responses to face blocks were compared to blocks of objects and scenes (faces > objects + scenes). Voxels that responded significantly more to faces over objects and scenes were defined as face-selective. Corresponding contrasts then defined object-selective (objects > faces + scenes) and scene-selective areas (scenes > faces + objects). We used family-wise error (FWE) correction to account for multiple comparisons.

**Figure 3:**
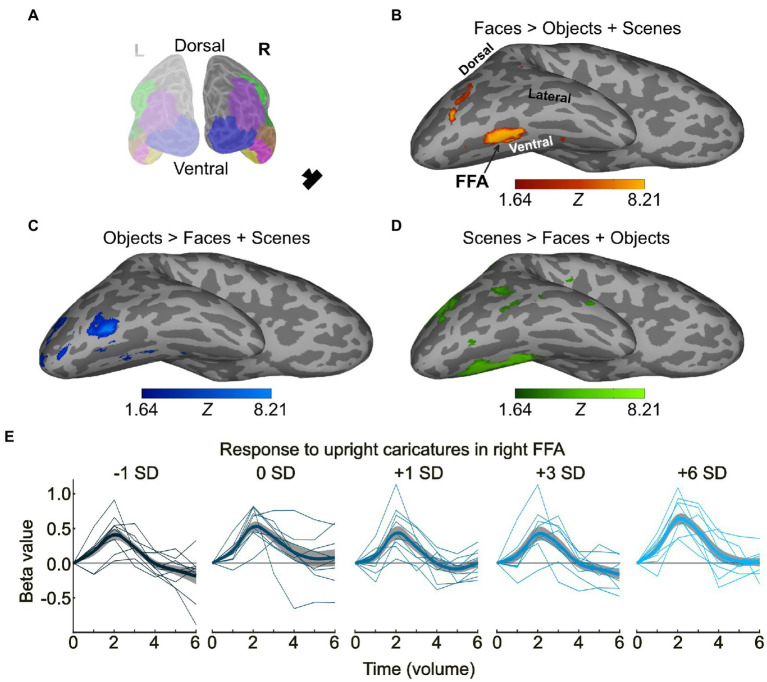
Defining face, object, and scene-selective regions and event-related fMRI responses in right FFA. **(A)** Posterior view of left and right inflated cortical hemispheres. Camera symbol indicates the view in panels B-D. Light grey, gyri; dark grey sulci. Regions in colour, Freesurfer parcellations used to form the bilateral occipitotemporal ROI, including lateral occipital (blue), fusiform gyrus (yellow), inferior temporal (pink), middle temporal (brown), bank of the superior temporal sulcus (dark green), inferior parietal (purple) and supramarginal gyrus (light green). **(B)** Face-selective regions in one participant based on the contrast faces > objects + scenes from the localiser scans (FFA, fusiform face area). Object-selective **(C)** and scene-selective **(D)** voxels defined using the contrasts objects > faces + scenes, and scenes > faces + objects, respectively. The colour bars in B-D show the Z-statistic for the contrast, thresholded at Z > 1.64 (corresponding to *p* < 0.05, with FWE correction). Maps show the voxels exclusively defined by these contrasts, with any overlap removed. **(E)** Response amplitude in the right FFA across participants from stimulus onset as a function of time for the five different levels of caricaturing (upright only) from the deconvolution analysis. Y values show the beta-coefficients from the deconvolution, normalised to *t* = 0. Thin lines show the average timeseries for each participant. Thick lines show the group average, smoothed over time (for display purposes only). Shaded areas show ± 1 SEM across participants. Solid grey line shows *Y* = 0. Colour represents the caricature level.

The functional ROIs were then defined on flat map representations of the corresponding statistical maps. A cluster corresponding to the FFA was present in each participant bilaterally (for details see Supplementary Table S4), however, in some participants, the boundaries were less clear, and even with family-wise error correction extended further along the fusiform gyrus and even into the neighbouring sulcus. In these cases, the FFA was defined as one contiguous cluster within a region restricted anatomically to the fusiform gyrus (from a FreeSurfer parcellation, FFA definition in each participant can be seen in Supplementary Figure S5). The pattern of response elsewhere however was more variable. Therefore, rather than trying to identify spatially consistent ROIs across participants, we simply allocated voxels to the 3 categories ‘face-selective’, ‘object-selective’ and ‘scene-selective’ based on the contrasts above. Face-selective voxels included the FFA. Voxels that responded significantly to more than one contrast were removed, such that each ROI only contained voxels that exclusively appeared for that contrast. Functional ROIs from one participant can be seen in Figures 3B–D.

#### 2.5.7. Univariate analysis

To assess the effect of caricature level in the FFA, and face-, object- and scene-selective areas, we first used a deconvolution analysis (e.g., see Gardner et al., 2005; Besle et al., 2013). This provided an estimate of the event-related BOLD response for each of the 7 stimulus types (5 caricature levels, upright images; 2 inverted images). From these event-related responses (see Figure 3E), we calculated an index of the response amplitude of the first 5 TRs after stimulus onset, by first normalising to the level at stimulus onset (the first TR) and then obtaining the mean signed deviation (MSD) across the subsequent four TRs.

#### 2.5.8. Multivariate pattern analysis

To look at patterns of response across the regions of interest, we also performed a correlation-based multivariate pattern analysis (MVPA). We compared the correlations in response patterns (beta values) between all 5 caricature levels of upright stimuli. The analysis was performed on the left and right FFA, left and right face-selective cortex, and left and right object-selective cortex.

The *β* values were obtained for each caricature scan repeat separately using a GLM similar to that described above, but assuming a canonical haemodynamic response function (double gamma). There were 5 explanatory variables (one for each upright caricature condition). The two additional conditions (inverted stimuli) were included as nuisance regressors. For each region of interest in the analysis, we then calculated the correlations of the *β* coefficient maps across regressors (avoiding within-scan comparisons). We then applied Fisher’s transform to convert from correlation, r, to Z and averaged these Z-values across scans for each participant separately.

## 3. Results

The average fMRI response in the FFA, as well as face-selective voxels overall, did not show a consistent change with either caricature level or inversion, as assessed by univariate analysis and ANOVA. Interestingly, however, in the right fusiform face area (FFA), we found that the patterns of fMRI response were more consistent as caricaturing increased as assessed by multivariate pattern analysis (MVPA). In contrast, object-selective regions showed an increase in both average fMRI response with increasing caricature level (univariate analysis), and the consistency of response pattern (MVPA).

### 3.1. Univariate analyses

To assess the effects of caricature level and inversion in the FFA we performed two separate within-subjects ANOVAs, one to assess the effect of caricature level and orientation using the least and most caricatured faces, and one to assess the effect of caricature level using all 5 caricature levels of upright stimuli. The first was a 2 × 2 × 2 ANOVA with hemisphere (left, right), stimulus orientation (upright, inverted) and caricature level (−1SD, +6SD), the second a 2 × 5 ANOVA with hemisphere (left, right) and caricature level (all 5 levels of upright caricature). ANOVAs were performed using IBM SPSS Statistics version 25.

To investigate the response amplitudes in the face-, object- and scene-selective voxels we performed the same two ANOVAs as for the FFA but including ROI as an additional independent variable with 3 levels (face-selective, object-selective, and scene-selective).

#### 3.1.1. Caricature level in the FFA

The event-related response profiles showed a clear trial-locked response to the 5 caricature levels across all regions. Figure 3E shows the average deconvolution timeseries for the right FFA across subjects (thick lines), as well as traces for individual participants (thin lines).

**Figure 4:**
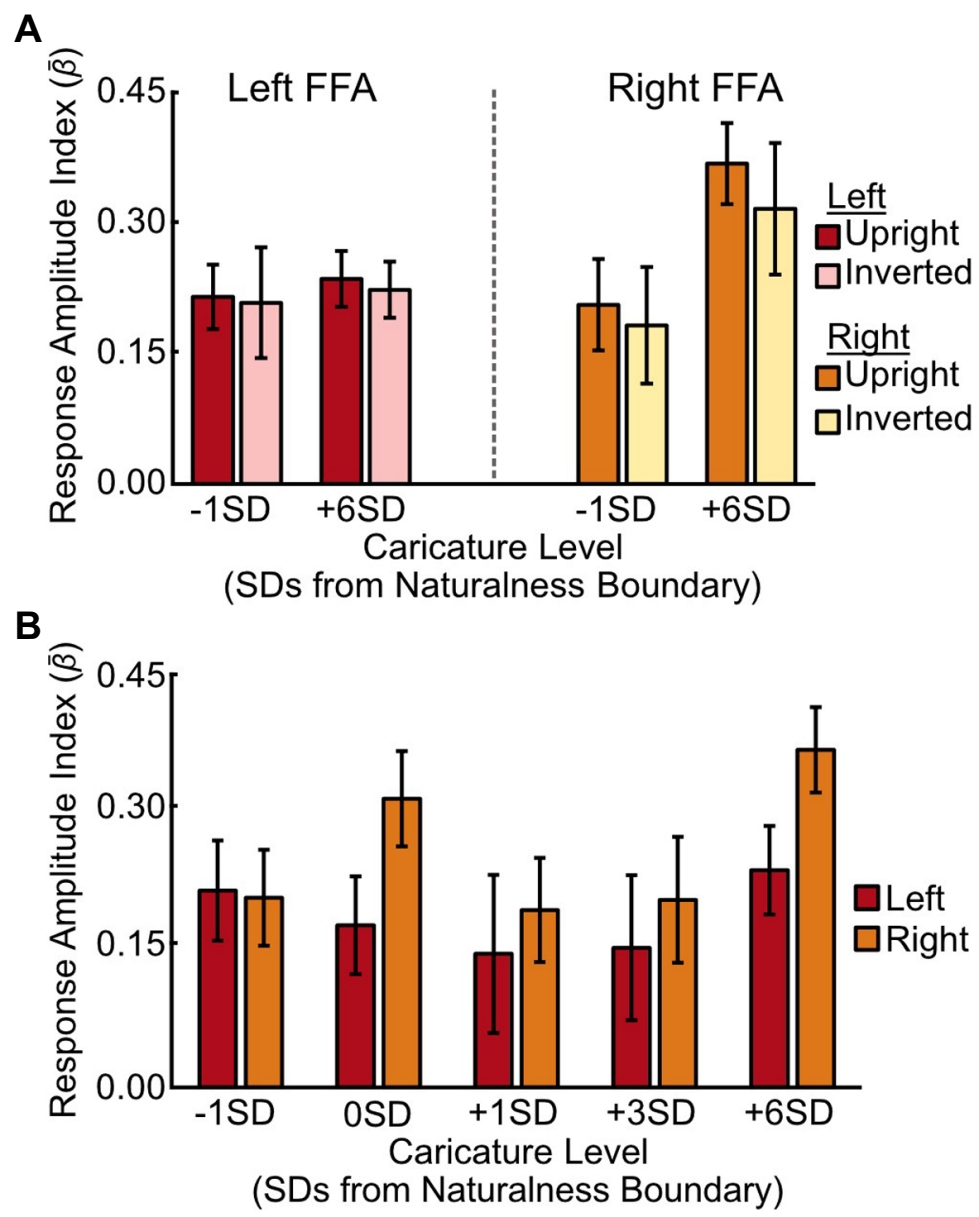
Response amplitudes in the FFA for different stimulus conditions. **(A)** Average response amplitude in the left (red/pink) and right (orange/yellow) FFA to the most (+6SD) and least (-1SD) caricatured faces in both the upright and inverted conditions. **(B)** Average response amplitudes in the left (red) and right (orange) FFA to each of the five caricature conditions for the upright stimuli only. Bars are grouped according to deviations from the average naturalness boundary (-1SD, closer to the average face; +6SD is highly caricatured). Y axes show the response amplitude index, measured by offsetting the *β* coefficients from the deconvolution analysis by *t*_0_, and averaging *t*_1-4_. Error bars show ± 1 SEM across participants.

When assessing caricature level (−1SD, +6SD), including both upright and inverted stimuli, there was no main effect of caricature level [*F*(1,8) = 3.08, *p* = 0.117, *η*_p_^2^ = 0.28], but there was a significant interaction between hemisphere and caricature level [*F*(1,8) = 5.86, *p* = 0.042, *η*_p_^2^ = 0.42]. The interaction was driven by a stronger increase in the response amplitude in the right FFA than the left FFA [*t*(8) = 2.42, *p* = 0.042] to an increase in caricature level (Figure 4A), although the effect of caricature level in the right FFA was marginal [*F*(1,8) = 5.18, *p* = 0.052, *η*_p_^2^ = 0.39].

We found no interaction between hemisphere and caricature level when we assessed all 5 levels of the upright stimuli [*F*(4,32) = 1.59, *p* = 0.200, *η*_p_^2^ = 0.17, Figure 4B], nor a main effect of caricature level [*F*(4,32) = 1.99, *p* = 0.119, *η*_p_^2^ = 0.20].

#### 3.1.2. Caricature level in face, object and scene-selective regions

**Figure 5:**
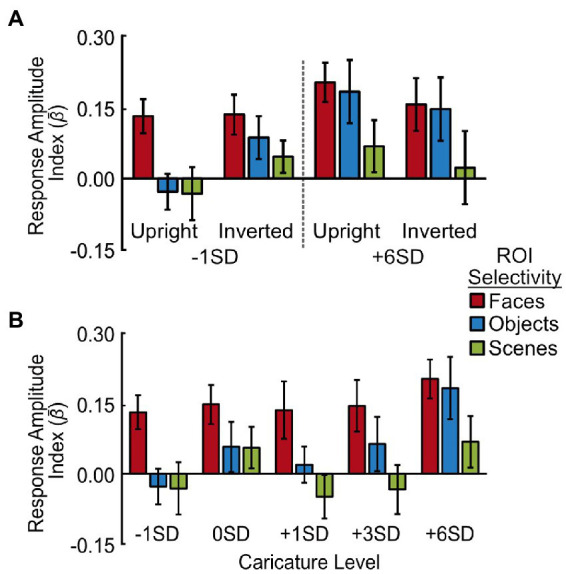
Response amplitudes to caricatured faces in face-, object- and scene-selective voxels. **(A)** Average response amplitudes for least (-1SD) and most (+6SD) caricatured stimuli. Data are grouped by image orientation (upright, inverted) and region of interest (face-, object-, and scene-selective voxels across hemispheres). **(B)** Average response amplitude to the 5 levels of caricatured, upright faces in face-selective (red), object-selective (blue) and scene-selective (green) voxels. As there was no interaction with hemisphere the response amplitudes are averaged across hemispheres. Y axes show the response amplitude index, measured by offsetting the *β* coefficients from the deconvolution analysis by *t*_0_, and averaging *t*_1-4_. Error bars show ± 1 SEM of the between-subjects variance.

We also compared responses across face-selective regions more generally, as well as in object- and scene-selective regions (see Supplementary Table S5 for details). The data are shown in Figure 5.

We found no significant main effect of caricature level when assessing the effect of caricature level (extremes) and inversion (Figure 5A), but there was a significant interaction with ROI [*F*(2,16) = 6.08, *p* = 0.011, *η*_p_^2^ = 0.43]. This interaction showed the effect of caricature level was only present in the object-selective cortex, with the object-selective cortex increasing in response amplitude with an increase in caricature level [*t*(8) = 2.49, *p* = 0.038].

When assessing all 5 levels of upright caricature, there was a main effect of caricature level [*F*(4,32) = 3.17, *p* = 0.027, *η*_p_^2^ = 0.28] driven by a general increase in response amplitude as a function of caricature level, which was particularly prominent for highly caricatured (+6SD) faces. The ANOVA showed there to be a positive linear trend between response amplitude and caricature level [*F*(1,8) = 16.83, *p* = 0.003, *η*_p_^2^ = 0.68]. Highly caricatured faces (+6SD) elicited a stronger response than -1SD [*t*(8) = 4.83, *p* = 0.001], 0SD [*t*(8) = 2.47, *p* = 0.039] and + 1SD [*t*(8) = 3.22, *p* = 0.012] caricatures, although only the first of these survived Bonferroni-correction (*a* = 0.005).

Although the interaction did not reach significance [*F*(8,64) = 1.96, *p* = 0.066, *η*_p_^2^ = 0.20], the overall effect was primarily driven by object-selective regions. The data in Figure 5B shows a constant response across caricature level in face-selective and scene-selective areas, but an increase in the response amplitude with increasing caricature level in the object-selective areas. To support this, separate ANOVAs for each ROI revealed that in the face-selective and scene-selective regions there was no effect of hemisphere, caricature, nor any interaction. In the object-selective regions there was no main effect of hemisphere nor interaction, but there was a significant effect of the caricature condition [*F*(4,32) = 4.76, *p* = 0.004, *η*_p_^2^ = 0.37] paired with a positive linear effect [*F*(1,8) = 26.69, *p* = 0.001, *η*_p_^2^ = 0.77]. Highly caricatured faces (+6SD) again elicited a stronger response over −1SD [*t*(8) = 5.31, *p* = 0.001], 0SD [*t*(8) = 2.82, *p* = 0.023] and + 1SD faces [*t*(8) = 3.97, *p* = 0.004]. The difference between +6SD and 0SD was not significant when correcting for multiple comparisons (*a* = 0.005). Interestingly faces on the naturalness boundary (0SD) also elicited a greater response than the most average (-1SD) faces [*t*(8) = 2.58, *p* = 0.032].

#### 3.1.3. Effects of ROI, orientation, and hemisphere

We found that there was a decrease in response amplitude from face, to object, to scene-selective cortex in response to our face stimuli. Main effects of ROI were significant when assessing the response to upright and inverted, −1SD and + 6SD caricatured stimuli [*F*(1.27,10.12) = 9.63, *p* = 0.008, *η*_p_^2^ = 0.55, Greenhouse–Geisser correction applied] and when assessing all 5 levels of upright stimuli [*F*(2,16) = 9.95, *p* = 0.002, *η*_p_^2^ = 0.55]. All pairwise comparisons were significant prior to correction (all *p* < 0.046) with the difference between face and scene-selective regions surviving correction (*a* = 0.017) in both analyses (both *p* < 0.012).

We found no main effects of, nor interactions with, orientation in any of our ROIs, and there were also no significant main effects of hemisphere. Generally, there was a greater response amplitude in the right hemisphere ROIs, which was most notable in the FFA when assessing the response to all five upright caricature levels [*F*(1,8) = 4.96, *p* = 0.057, *η*_p_^2^ = 0.38].

### 3.2. Multivariate pattern analysis

**Figure 6:**
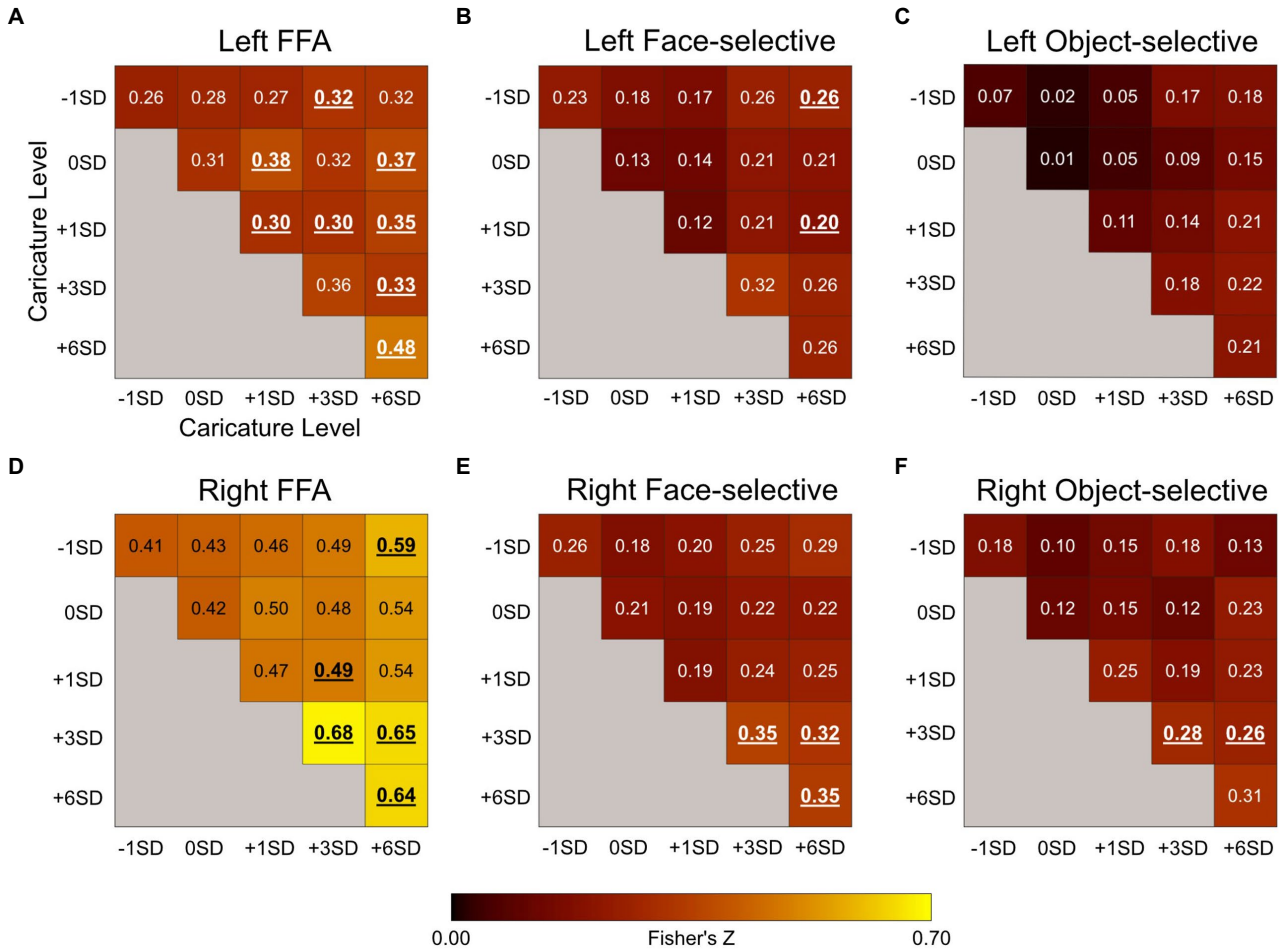
Plots showing the Fisher’s Z for the correlation coefficients between *β* maps corresponding to the 5 upright levels of caricature from the MVPA analysis in the left FFA **(A)**, the left face-selective voxels **(B)**, left object-selective voxels **(C)**, the right FFA **(D)**, right face-selective voxels **(E)**, and the right object-selective voxels **(F)**. The diagonal reflects the average correlations between the response patterns to stimuli of the same caricature level, whilst the off-diagonal reflects correlations between different caricature levels. Only between-scan correlations were assessed. Values in bold and underlined were significantly greater than 0 at a group level, measured using one-sample *t*-tests (Bonferroni-corrected *a* = .003̇). Font colour for display purposes only.

The results of the correlation analysis can be seen in Figure 6. In each ROI, we tested whether there was a significant positive correlation in the response patterns for each pair of caricature levels. Significance was assessed using one-sample t-tests to test if the group-level Z-value was significantly greater than 0 (Bonferroni-corrected *a* = .003) and is indicated by bold, underlined values in Figure 6. We then assessed how the response patterns varied as a function of caricature level using a one-way within-subjects ANOVA with the 5 levels of ‘same’ caricature correlations (i.e., the diagonals in Figure 6) as the independent variable.

#### 3.2.1. Caricature level in the FFA

In the right FFA, the correlation coefficient (converted to Fisher’s Z) increased as a function of caricature level (Figure 6D), supported by a significant positive linear trend [*F*(1,8) = 7. 60, *p* = 0.025, *η*_p_^2^ = 0.49], indicating increasing consistency in the patterns of responses between stimulus categories including highly caricatured faces. In the left FFA, many of the correlations were significant at a group level, but the overall increase with caricature level, as seen in the right FFA, was not.

#### 3.2.2. Caricature level in face and object-selective cortex

When looking at the consistency of response patterns in face and object-selective regions more broadly, we found that only object-selective regions bilaterally showed an increase in consistency with caricature level. Right face-selective regions were sensitive to caricature level, but the change in response pattern was less clear.

In the left face-selective regions there was no significant effect of caricature level on the correlations. In the right face-selective cortex there was a significant main effect of caricature level [*F*(4,32) = 5.06, *p* = 0.003, *η*_p_^2^ = 0.39] however the response profile was less clear than in the right FFA.

In both the left and the right object-selective regions there was a positive linear trend in the correlation to same caricature level trials as a function of caricature level [left: *F*(1,8) = 21.91, *p* = 0.002, *η*_p_^2^ = 0.73; right: *F*(1,8) = 10.09, *p* = 0.013, *η*_p_^2^ = 0.56]. At the group level however, no Z-values were greater than 0 in the left object-selective cortex. In the right hemisphere only correlations between +3SD and + 3SD stimuli and + 3SD and + 6SD stimuli were significant.

## 4. Discussion

We investigated the effect of caricaturing on the fMRI response in visual areas defined by preference to faces, objects, and scenes. Based on evidence of ramp coding in single cell recordings in macaques (Leopold et al., 2006; Chang and Tsao, 2017) to face stimuli of increasing distance from the mean face in the neuron’s preferred direction of change, we reasoned that there may be an increase in the response amplitude of the FFA with increasing caricature level, even when faces appeared heavily distorted.

Surprisingly, we found no clear change in the average response amplitude in the FFA, or face-selective cortex more broadly, with increasing caricature level. In contrast, we found an increase in response in object-selective cortex, particularly for highly caricatured faces. There was no significant change in response in scene-selective areas.

An increase in the consistency of the response pattern in object-selective cortex was also observed with increasing caricature level, measured using MVPA. Caricaturing therefore both enhanced the average response, and the consistency in which the stimuli were processed within object-selective cortex. How or why caricatured faces activate object-selective cortex is unclear.

The results seen in object-selective regions may result from changes to low-level or even mid-level properties that vary with caricature level, rather than a response to caricatured faces *per se* or the assignment of hyper-caricatured faces to a separate object class other than faces. Higher-level visual regions, including the FFA (Weibert et al., 2018), are sensitive to the lower-level image properties that are characteristic of different categories of objects (see Andrews et al., 2015). Caricaturing may have therefore emphasised particular low or mid-level properties that object-selective neurons are tuned to, such as certain shapes or curvatures that distinguish animate faces, bodies and animals from inanimate objects (Zachariou et al., 2018; Yue et al., 2020; Yetter et al., 2021) or changes in bilateral symmetry (Bona et al., 2015). The areas defined as object-selective responded *more* to objects than faces and scenes despite many voxels responding to all three categories (see Supplementary Figure S6) so the changes with caricature level may have generated stimulus properties that are more characteristic of generic objects than faces. The changes in our stimuli, including changes in texture, colour (Lafer-Sousa et al., 2016), shape, curvature (Yue et al., 2020; Yetter et al., 2021) and external contours, as well as higher-level changes, may have caused a shift in object-space (Bao et al., 2020).

Regardless of the exact mechanism for why or how caricatures activate object-selective cortex, it is evident that object-selective cortex is sensitive to caricature level, raising the possibility of its involvement in the perceptual evaluation of faces. To our knowledge, these are the first findings that show that caricatured faces elicit increased responses in regions typically involved in processing objects. These findings can potentially have important implications for understanding how we might form impressions from or recognise more distinctive faces. Although the faces in our experiment were artificially caricatured, faces in the real world can be naturally distinctive too, for example a number of (often genetic) disorders give rise to naturally distinctive faces (Gad et al., 2008; Babovic-Vuksanovic et al., 2012; Dolci et al., 2021). Our findings therefore raise a number of questions as to whether, and if so how, object-selective cortex contributes to our social evaluation of faces.

Returning to face-selective cortex, we initially found evidence that our most caricatured faces elicited a stronger response in the right FFA (but not left) compared to our least caricatured faces when we included both upright and inverted stimuli. This interaction between hemispheres is potentially consistent with the idea of a greater involvement of the right FFA in face perception, for example, electrical brain stimulation only impacts perception of faces when applied to the right FFA and not the left (Rangarajan et al., 2014).

We found no evidence of an effect of caricature level in face-selective cortex however when we assessed for a graded change in response amplitude across the complete range of caricature levels; there was no effect of hemisphere, caricature level nor an interaction, in either the FFA, specifically, or face-selective regions more widely. This may reflect a plateau in the BOLD response (Loffler et al., 2005; McKone et al., 2014; Carlin and Kriegeskorte, 2017). Since most of the stimuli were ‘caricatured’ to some degree the results could reflect response saturation; even the least caricatured stimuli could be identified as a particular individual. Alternatively, since Chang and Tsao (2017) report ramp-like tuned cells which increase their firing along an axis passing through the mean face, increasing caricature level may increase the firing of some cells whilst reducing the firing of others, leading to no net increase in the response across the population within a voxel.

Interestingly, the multivariate pattern of response in the right FFA became more consistent with increasing caricature level. This suggests a pattern of systematic increases and decreases in response rate across a population of cells. For the right face-selective regions more broadly, the change in spatial consistency was less clear, with slightly increased consistency for more caricatured faces, but decreased spatial consistency for intermediate caricatures. In the left hemisphere there was no effect of caricature level, consistent with the functional differences in the left and right FFA. The increase in spatial consistency in the absence of an average increase in the fMRI response in the right FFA is particularly interesting since stimuli at the same caricature level could vary substantially in terms of their low-level properties, given that different PCA components were modulated. Despite these low-level differences, which became more pronounced as caricaturing increased, the response pattern became significantly more consistent. This indicates that the increase in the overall consistency of the pattern was maintained regardless of any variation in the individual patterns themselves. Likewise, the general increase in consistency appeared to hold regardless of whether we compared the response patterns between the same caricature level, or different caricature levels. This suggests that different levels of caricature are processed by the same set of voxels, and is consistent with the idea that voxel-wise responses scale with varying distances from the average in a face space.

Our analysis of inversion showed no effect of orientation nor interaction with it. The lack of effect of orientation is at odds with some prior research showing an inversion effect in FFA (Yovel and Kanwisher, 2004, 2005; Nasr and Tootell, 2012; James et al., 2013), but is in line with other findings in literature suggesting that the inversion effect is weak (Gilaie-Dotan et al., 2010) or even absent (e.g., see Aguirre et al., 1999; Haxby et al., 1999; Epstein et al., 2006). The initial evidence of an effect of caricature level alongside a lack of inversion effect potentially suggests that the FFA is sensitive to changes in low-level properties, consistent with prior evidence (Weibert et al., 2018) and that this may not be specific to upright faces.

To conclude, in the FFA and face-selective areas more generally, we found no substantive effect of caricature level on the average response amplitude, although we did find evidence that the right FFA is sensitive to caricature level using MVPA, with the consistency of the response pattern increasing with caricature level. In contrast, we found a significant increase in both the response pattern consistency and the average response amplitude in object-selective cortex to increasing caricature level. This suggests that caricatured faces might recruit cortex typically defined as object-selective, potentially because they share more low-level features with objects. This may have implications for understanding how distinctive faces might be processed, both in terms of recognition and forming impressions.

## Data availability statement

The datasets presented in this study can be found in online repositories. The names of the repository/repositories and accession number(s) can be found at: “Investigating the fMRI response to highly caricatured faces,” doi:10.17605/OSF.IO/JEC6U.

## Ethics statement

The studies involving human participants were reviewed and approved by School of Psychology Ethics Committee, University of Nottingham. The patients/participants provided their written informed consent to participate in this study. Written informed consent was obtained from the individual(s) for the publication of any potentially identifiable images or data included in this article.

## Author contributions

RE, AJ, and DS contributed to the conception and design of the study. RE built the experimental scripts, wrote the manuscript. RE and DS collected the data. RE analysed the data with support from DS and AJ. AJ and DS supervised the study. All authors contributed to manuscript revision, read, and approved the final version for submission.

## Funding

The scan time was awarded by the Sir Peter Mansfield Imaging Centre through a pump-priming scheme. This work was also supported by the Economic and Social Research Council [grant number: ES/P000711/1] for providing inconvenience allowances for participants. Open access fees were supported by the UKRI block grant for the University of Nottingham.

## Conflict of interest

The authors declare that the research was conducted in the absence of any commercial or financial relationships that could be construed as a potential conflict of interest.

## Publisher’s note

All claims expressed in this article are solely those of the authors and do not necessarily represent those of their affiliated organizations, or those of the publisher, the editors and the reviewers. Any product that may be evaluated in this article, or claim that may be made by its manufacturer, is not guaranteed or endorsed by the publisher.
