# Supplementary material for: fMRI evidence that hyper-caricatured faces activate object-selective cortex
Source: Front Psychol. 2023 Jan 12;13:1035524. doi: 10.3389/fpsyg.2022.1035524 (PMC9878608; doi:10.3389/fpsyg.2022.1035524)
Supplement: Supplementary file 1 [file Data_Sheet_1.DOCX]

**Note to accompany the supplementary videos.**

The videos show the animation that was shown to the participant at the start of each trial in the behavioural experiment. On each trial one principal component was manipulated, and the videos show examples of the animations for each component. The face starts close to average, is heavily caricatured, and then returns to average. The maximal amount of caricaturing varies across trials, so on some trials the face will become slightly more caricatured that on other trials. The aim of these animations is to show the full range of caricaturing for the trial. This also serves to pre-expose participants with heavily caricatured stimuli before the fMRI scans.
