# Supplementary material for: fMRI evidence that hyper-caricatured faces activate object-selective cortex
Source: Front Psychol. 2023 Jan 12;13:1035524. doi: 10.3389/fpsyg.2022.1035524 (PMC9878608; doi:10.3389/fpsyg.2022.1035524)
Supplement: Supplementary file 2 [file Image_1.PDF]

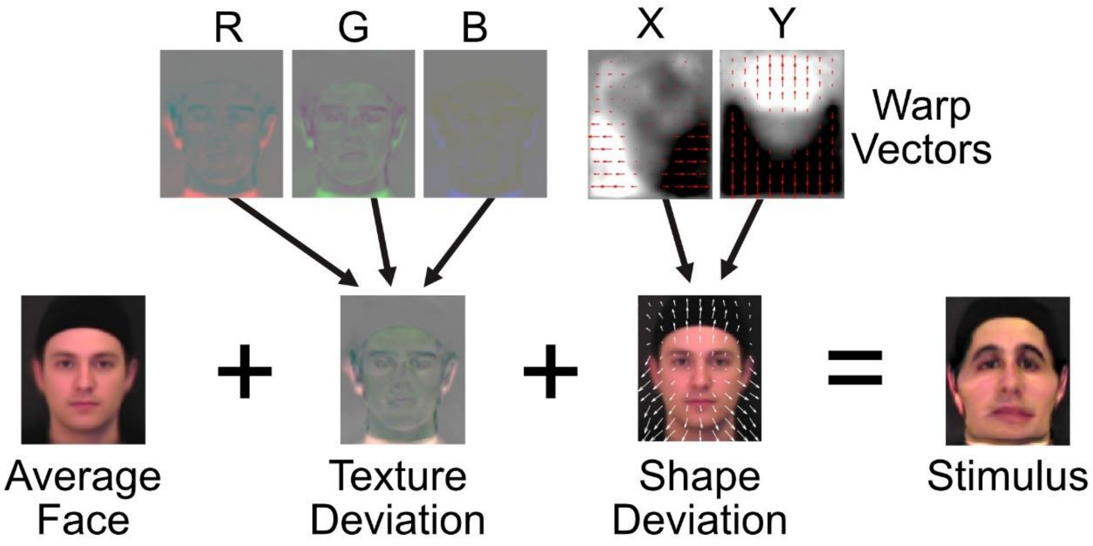

Supplementary Figure 1. Creating stimuli by adding deviation vectors to the origin of face space. Stimuli were created by adding texture and shape deviations to the image corresponding to the origin of the PCA-based face space. The texture deviation stores how much of each RGB channel is to be added to the average face for each pixel. Note that for easier visualisation the RGB changes have been exaggerated here. The x-y warp fields contain the horizontal (x) and vertical (y) pixel displacements necessary to distort the average face shape. Lighter areas show leftward and upward displacements, while darker areas show rightward and downward displacements.
