# Supplementary material for: fMRI evidence that hyper-caricatured faces activate object-selective cortex
Source: Front Psychol. 2023 Jan 12;13:1035524. doi: 10.3389/fpsyg.2022.1035524 (PMC9878608; doi:10.3389/fpsyg.2022.1035524)
Supplement: Supplementary file 3 [file Image_2.PDF]

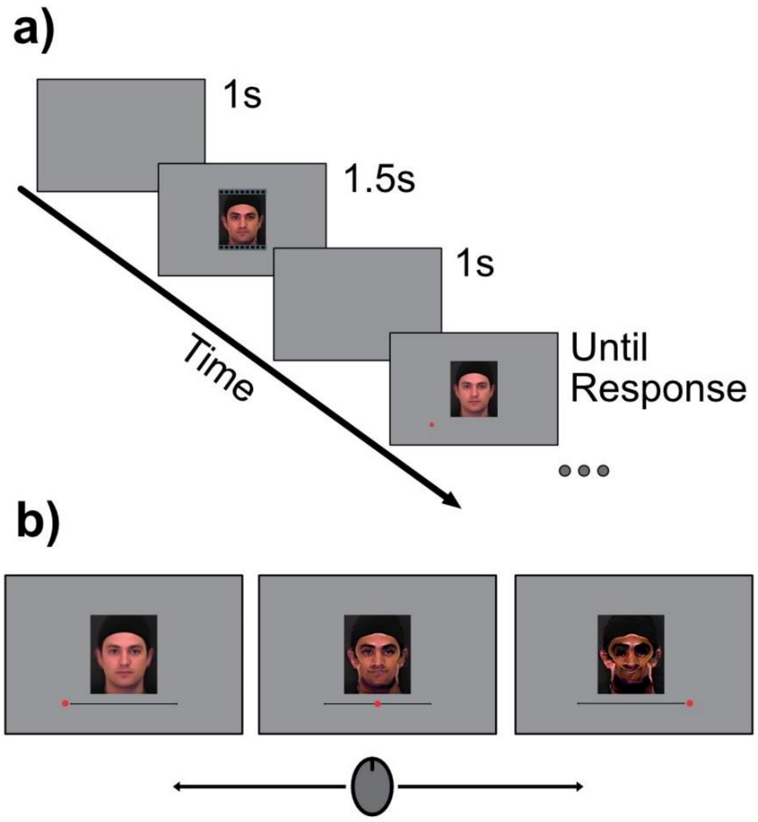

Supplementary Figure 2. Depiction of behavioural experiment. Trial timings (**A**) showing an inter-trial interval of 1 second, followed by the demonstration video lasting 1.5 seconds, followed by a 1 s inter-stimulus interval before the appearance of the manipulable stimulus. The demonstration video starts with a close to average face that becomes heavily caricatured and then returns to average. (**B**) The manipulable stimulus is presented until response and can be manipulated by the participant to appear more or less average by moving the mouse left to right respectively. When the participant has found the boundary between natural/physically plausible and unnatural/physically implausible the participant responds by clicking either key on the mouse.
