# Supplementary material for: fMRI evidence that hyper-caricatured faces activate object-selective cortex
Source: Front Psychol. 2023 Jan 12;13:1035524. doi: 10.3389/fpsyg.2022.1035524 (PMC9878608; doi:10.3389/fpsyg.2022.1035524)
Supplement: Supplementary file 4 [file Image_3.PDF]

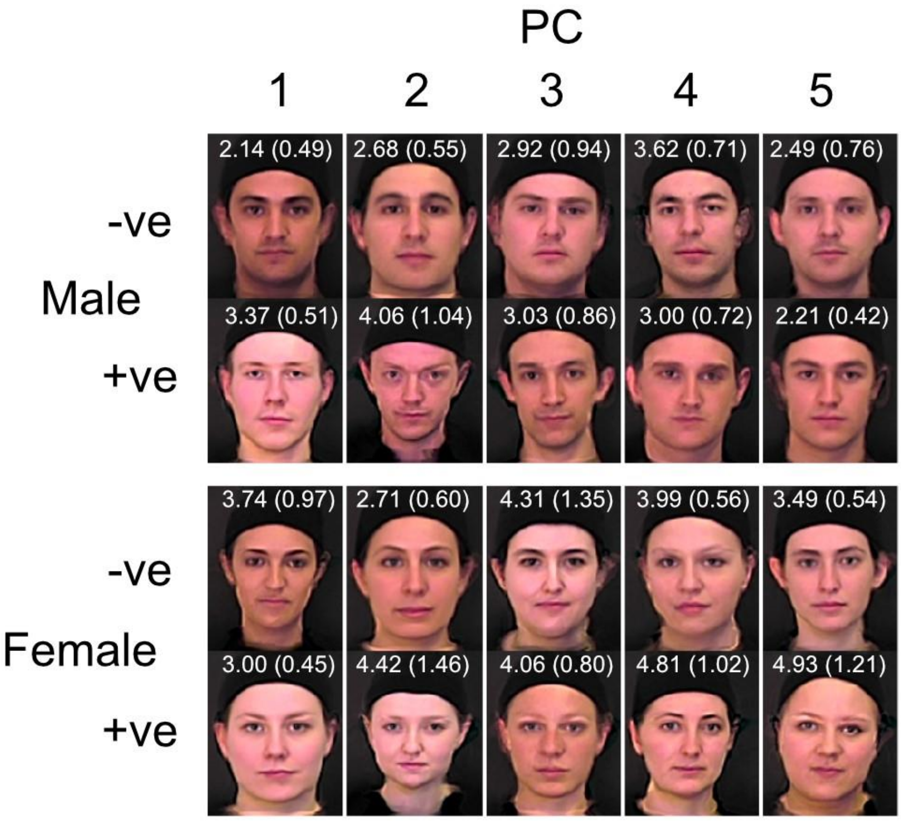

Supplementary Figure 3. Group average naturalness boundaries. Stimuli representing the average transition point for the first seven participants between natural, physically plausible and unnatural, physically implausible. Rows, two directions (sign) in PCA space for each gender. Columns, five principal components scaled according to the transition points. Numbers, average transition point in terms of the number of standard deviations in PCA space (with respect to the input data) along the given component. Numbers in parentheses, the between-subjects standard deviation from the behavioural results.
