# Supplementary material for: fMRI evidence that hyper-caricatured faces activate object-selective cortex
Source: Front Psychol. 2023 Jan 12;13:1035524. doi: 10.3389/fpsyg.2022.1035524 (PMC9878608; doi:10.3389/fpsyg.2022.1035524)
Supplement: Supplementary file 5 [file Image_4.PDF]

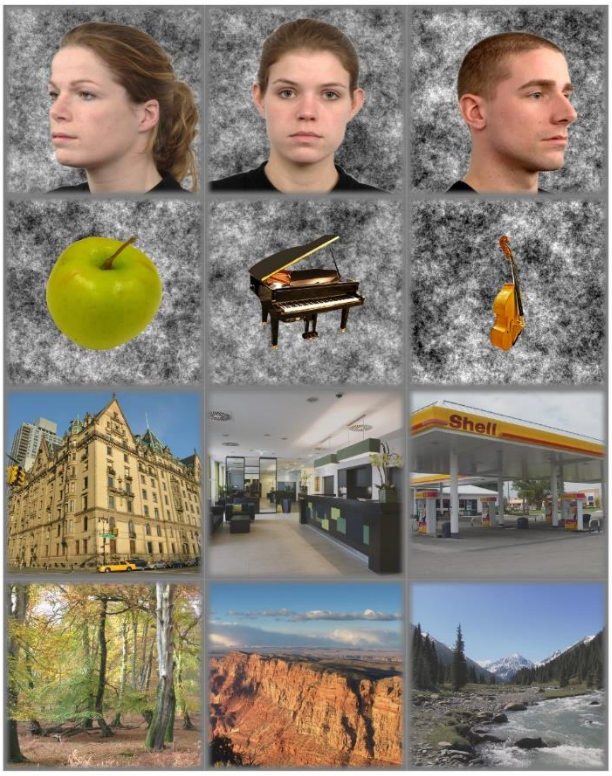

Supplementary Figure 4. Example stimuli for the localiser scan showing faces, objects, manmade scenes and natural scenes. Manmade and natural scenes formed one block type encompassing scenes as a whole.
