# Supplementary material for: fMRI evidence that hyper-caricatured faces activate object-selective cortex
Source: Front Psychol. 2023 Jan 12;13:1035524. doi: 10.3389/fpsyg.2022.1035524 (PMC9878608; doi:10.3389/fpsyg.2022.1035524)
Supplement: Supplementary file 6 [file Image_5.PDF]

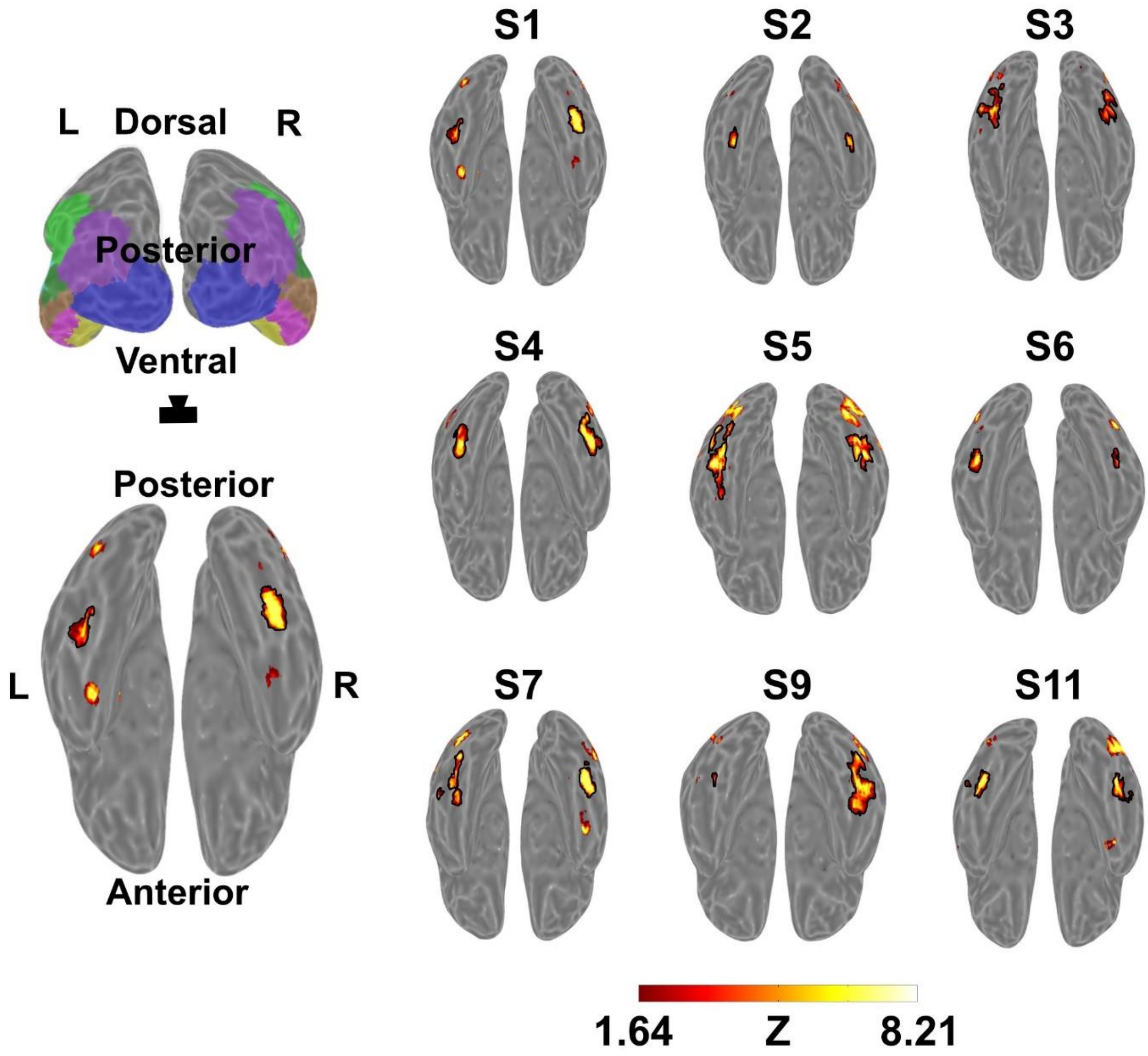

Supplementary Figure 5. Defining the FFA in all participants. The two leftmost images depict the current view, with images taken from underneath the ventral surface. The left and right image of each pair show the left and right hemispheres respectively. The images S1-S9 show the FFA definition in each participant (the region surrounded by the black border). The statistical maps show the face-selective regions defined by the contrast faces > objects + scenes that survived FWE correction. Maps show the z-values of the contrasts.
