# Supplementary material for: fMRI evidence that hyper-caricatured faces activate object-selective cortex
Source: Front Psychol. 2023 Jan 12;13:1035524. doi: 10.3389/fpsyg.2022.1035524 (PMC9878608; doi:10.3389/fpsyg.2022.1035524)
Supplement: Supplementary file 7 [file Image_6.PDF]

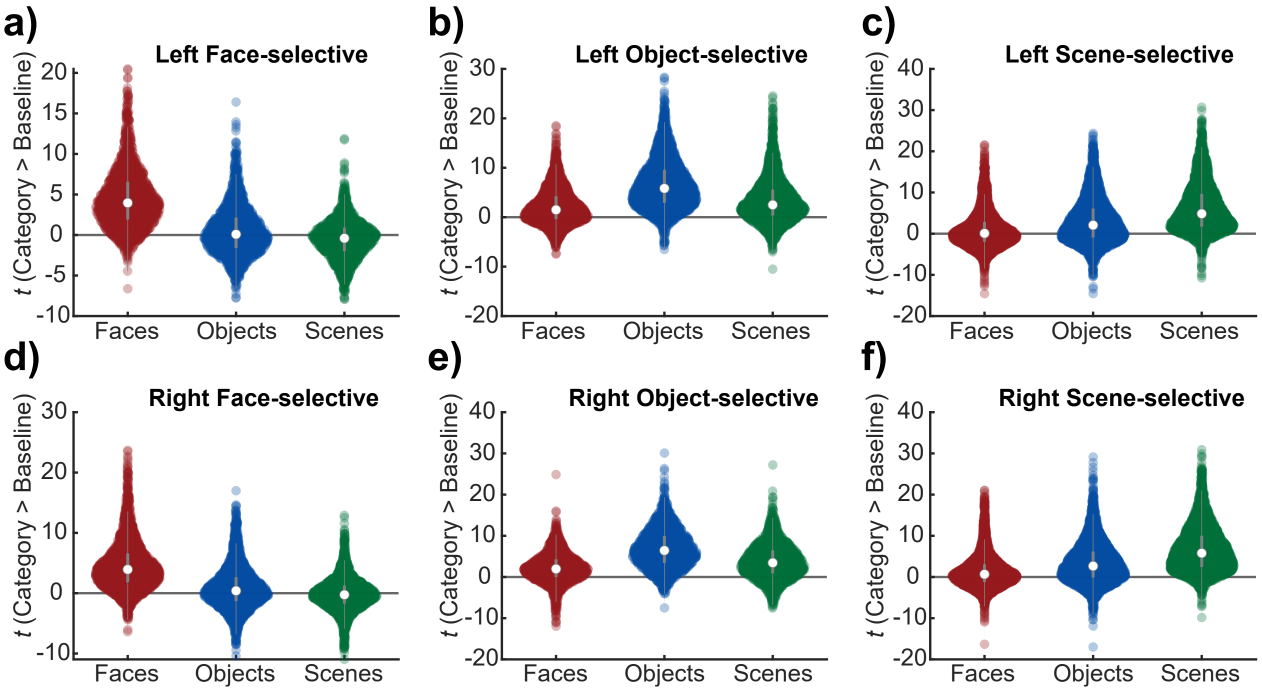

Supplementary Figure 6. Violin plots to show the distribution of responses to faces (red), objects (blue) and scenes (green) compared to baseline (t-value of the contrast). (**A-C**) The responses in the face-selective, object-selective and scene-selective voxels of the left temporal cortex. (**D-F**) The responses in the right temporal cortex. Above the zero-line show greater responses to the stimuli than baseline, beneath show inhibited responses to the stimuli compared to baseline. ROIs were defined by contrasting the response between different stimulus classes, e.g., face-selective was defined as voxels responding significantly more to faces than objects and scenes. Voxels that responded significantly in more than one of these contrasts were excluded.
